# Association Between Visit-to-Visit Glucose Variability and Brain Morphology and Cognitive Function in Type 2 Diabetes

**DOI:** 10.1210/clinem/dgaf228

**Published:** 2025-04-08

**Authors:** Weiye Lu, Die Shen, Shijun Qiu

**Affiliations:** First Clinical Medical College, Guangzhou University of Chinese Medicine, Guangzhou 510000, China; First Clinical Medical College, Guangzhou University of Chinese Medicine, Guangzhou 510000, China; Department of Radiology, The First Affiliated Hospital of Guangzhou University of Chinese Medicine, Guangzhou 510000, China; State Key Laboratory of Traditional Chinese Medicine Syndrome, Guangzhou 510000, China

**Keywords:** glucose variability, cognitive function, brain morphometry, type 2 diabetes mellitus

## Abstract

**Objective:**

To investigate the effect of varying visit-to-visit glucose variability (GV) on brain morphometry and cognitive performance in patients with type 2 diabetes mellitus (T2DM).

**Methods:**

This was a retrospective cohort study in which we recruited 426 participants (173 T2DM patients and 253 healthy controls) who underwent cognitive assessment and structural magnetic resonance imaging. In patients with T2DM, visit-to-visit GV was calculated using the SD of glycated hemoglobin (HbA1c) during the follow-up period. Multiple linear regression models were used to analyze the associations between different levels of GV and brain morphometry as well as cognitive function after adjusting for mean HbA1c levels and other traditional risk factors.

**Results:**

Higher GV is associated with poorer global cognitive performance and executive function. After full multivariate adjustment, higher GV is linked to cortical thinning in the left superior parietal cortex, right postcentral gyrus, and insula, as well as a reduction in total gray matter volume. In contrast, no association was observed between GV and cortical volume or surface area.

**Conclusion:**

Our findings indicate that higher visit-to-visit GV is associated with reduced cortical thickness, total gray matter volume atrophy, and poorer cognitive performance in patients with T2DM, and these associations are independent of mean HbA1c levels.

Type 2 diabetes mellitus (T2DM) is one of the primary causes of increased risk of vascular, renal, and neurological complications (1). The prevalence of diabetes in China continues to rise year by year, with 1 in 9 adults suffering from diabetes and more than a third of Chinese adults suffering from prediabetes (2). Because of aging and the increasing prevalence of obesity, the incidence of diabetes is expected to continue to increase. Fasting blood glucose and HbA1c measurements are commonly used to assess blood glucose control and diagnose diabetes, but they do not reflect fluctuating levels of blood glucose, known as glucose variability (GV) (3). The link between GV and cognitive decline has received increasing attention from physicians. Recent studies indicate that GV levels correlate with cognition in patients with T2DM and may be an accelerator or risk factor for the onset and progression of cognitive impairment (4). An animal study found that elderly T2DM rats with glucose fluctuations exhibited poorer cognitive performance compared to the control group (5). A population-based study that defined GV using SD, average real variability, and variability independent of the mean reported that higher GV is associated with modest global cognitive decline in participants with T2DM (6). The process via which elevated glycemic variability contributes to diabetes-associated cognitive decline may involve heightened generation of reactive oxygen species, subjecting blood vessels to oxidative stress and thus harming the central nervous system (CNS) (7, 8).

However, among the articles exploring the association between GV and cognitive function, limited research has investigated the association between varying levels of GV and structural brain magnetic resonance imaging (MRI). Therefore, our study aims to investigate the characteristic associations between visit-to-visit GV, structural brain MRI, and cognitive function. We hypothesized that T2DM patients with high GV demonstrate more severe structural brain abnormalities and poorer cognitive function.

## Methods

### Study Population

This study randomly selected 358 patients with T2DM who regularly visited the endocrinology clinic at the First Affiliated Hospital of Guangzhou University of Chinese Medicine between 2019 and 2023. Outcome variables, including cognitive assessments and brain MRI, were obtained during their last visit. Inclusion criteria for patients with T2DM were as follows: (i) T2DM patients were diagnosed according to the 2021 American Diabetes Association statement (9), including those with self-reported diabetes previously diagnosed by a physician and those currently using antidiabetic medications; (ii) age between 30 and 70 years; (iii) right-handedness; and (iv) a minimum duration of diabetes of at least 1 year and at least 3 available glycate hemoglobin (HbA1c) data. The following were the exclusion criteria: (i) contraindications to MRI examination; (ii) previous diagnosis of definite stroke, dementia, Parkinson's and other diseases affecting cognitive function; (iii) diseases seriously affecting the quality of the body, such as severe heart disease (defined as a history of myocardial infarction within the past 6 months, unstable angina, or New York Heart Association (NYHA) Class III-IV heart failure), tumors, transient ischemic attack, patients with chronic infections and psychiatric disorders; and (iv) patients with head magnetic resonance examination showing extensive white matter demyelination (WMH Fazekas visual rating scale ≥2). Among the 358 T2DM patients initially selected at random, 173 met the inclusion criteria, with a follow-up duration of 2.7 ± 0.6 years. During the same period, 253 healthy controls (HCs) were recruited from the community through poster advertisements, and cognitive assessments and brain MRI results were also obtained. The inclusion and exclusion criteria for the HCs were consistent with those for the T2DM patients, except that they did not have diabetes and only had 1 available HbA1c measurement. The flowchart for the inclusion of the study population is shown in Supplementary Fig. S1 (10).

The committees of the First Affiliated Hospital of Guangzhou University of Chinese Medicine approved this study, and all participants gave written informed consent.

### Measurement of Visit-to-Visit GV

The T2DM patients ultimately included in the analysis visited the endocrinology outpatient clinic or inpatient department of our hospital at least once every 3 months between 2019 and 2023, with at least 3 available HbA1c measurements. During each visit, participants were required to have fasted for at least 8 hours before venous blood was drawn in the morning. Additionally, they were instructed to temporarily discontinue oral hypoglycemic medications or insulin before the blood draw. HbA1c measurements were recorded during each visit over this period. Based on the HbA1c records from each visit, the SD of HbA1c was used to assess GV in the T2DM patients (11).

### Cognitive Assessment

Evaluators were rigorously trained to administer uniform cognitive tests for each subject without knowledge of the subject's medical history and grouping. The cognitive tests cover diverse domains of cognition, including the following: (i) the Mini-Mental State Test (MMSE) to assess general cognitive function (12); (ii) the Auditory Verbal Learning Test (AVLT) to assess instantaneous memory and delayed recall (13); (iii) the Digit Symbol Substitution Test (DSST) to assess attention and information processing speed (14); (4) the Digital Span Test (DST) to assess attention and working memory (15); and (5) the Grooved Pegboard Test (GPT) (16) and Trail Making Test A (TMT-A) (17) to assess executive function.

### Assessment of Brain Morphometry

**Figure 1.**
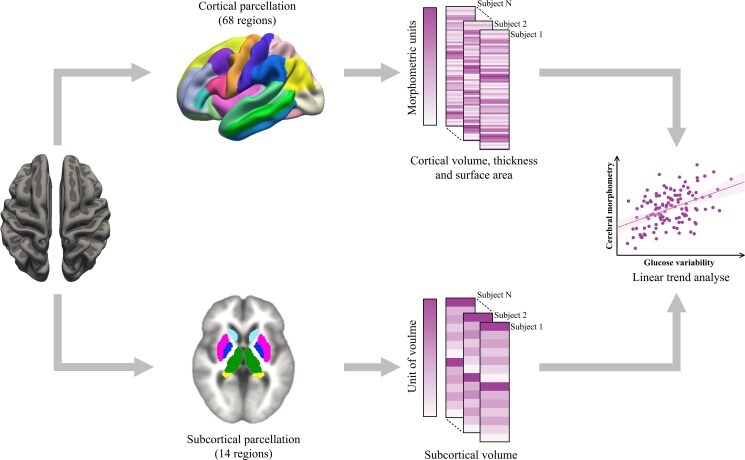
Workflow for analyzing cerebral morphometry. Cortical gray matter was divided into 68 cortical brain regions using the Desikan-Killiany atlas, and cortical volume, thickness, and surface area were calculated. In addition, total gray matter volume, total white matter volume, and the volumes of 14 subcortical brain regions were calculated. Finally, linear trend analysis of brain morphometry was analyzed using regression models.

MRI data were acquired using a 3.0-T MRI scanner (Prisma, Siemens) with a 64-channel head coil. T1-weighted magnetization-prepared rapid gradient echo sequences and fluid-attenuated inversion recovery sequences were used, with the latter employed to exclude participants with extensive white matter demyelination. Further details on the imaging methodology have been reported elsewhere (18). T1 images of all participants were processed using the recon-all function of FreeSurfer v7.3.2. The recon-all function is a fully automated processing pipeline designed for cortical and subcortical segmentation, surface reconstruction, and morphometric quantification of structural MRI data. This automated preprocessing method has been described in greater detail elsewhere (19). Based on the Desikan-Killiany atlas (20), the cortex was parcellated into 68 brain regions, and 3 cortical morphometric outcome variables (gray matter volume, cortical thickness, and surface area) were calculated. Additionally, this study included other brain volume outcome variables, such as total white matter volume, total gray matter volume, and subcortical gray matter volume (defined as the total volume of the hippocampus, thalamus, amygdala, putamen, nucleus accumbens, caudate nucleus, and pallidum). After image preprocessing, a researcher checked the segmentation (eg, white matter surfaces, gray-white matter boundaries) and registration accuracy of all results using FreeSurfer's Freeview application. The workflow for analyzing brain morphometry is shown in Fig. 1.

### Statistical Analysis

Patients with T2DM were divided into tertiles based on the SD of visit-to-visit HbA1c. Continuous variables used linear regression and categorical variables used Cochran–Armitage trend test to determine whether the trends in the characteristics of the study population were significant. If the population characteristics meet the criteria for continuous variables, they are presented as mean ± SD; otherwise, they are expressed as percentages. Multivariable linear regression models were used to examine the association between varying levels of GV and cognitive function as well as brain morphometry. In the linear trend analysis, the categorical variable “GV” (HCs = 0, low GV = 1, medium GV = 2, high GV = 3) was used in the regression model, with the healthy control group serving as the reference group. The main results are presented as unadjusted β coefficients with their 95% CI. The β estimates represent the change in units of each outcome per one tertile increase in GV. Three regression models were fitted: Model I adjusted for baseline age, sex, years of education, and intracranial volume. Model II additionally adjusted for body mass index, the ratio of total to high-density lipoprotein (HDL) cholesterol, alcohol consumption (yes or no), smoking status (current/former vs never), serum triglycerides, and hypertension (yes or no). Model III included all covariates from Model II and further added mean HbA1c. To mitigate the risk of Type I errors in interpreting the association between GV and brain morphometry due to multiple comparisons, we applied the False Discovery Rate (FDR) correction, with associations deemed significant at an FDR threshold of ≤0.05. Additionally, we used Model III to test the interaction between GV and sex to evaluate potential effect modification by sex. In cases where significant interactions were observed (*P*_interaction_ < .10 after FDR correction), we conducted stratified analyses. All statistical analyses were completed using SPSS (IBM, SPSS, version 27).

### Sensitivity Analysis

Three sensitivity assessments were conducted. First, to explore whether abnormal brain volume affects the stability of the results, individuals with intracranial volume outside the range of 3 SDs were excluded. Second, due to the lack of a suitable standard measure for visit-to-visit GV, we used the coefficient of variation (CV) of HbA1c to assess GV in patients with T2DM. The CV (%) is calculated by taking the SD as a percentage of the mean: CV% = SD/mean × 100. Finally, given that participants with prediabetes may experience subclinical glucose fluctuations, potentially leading to biased estimates of the association between GV and brain morphology, we identified and excluded individuals with prediabetes from the HCs in accordance with the 2021 American Diabetes Association guidelines to ensure these participants were not included in the analysis.

## Results

### General Characteristics

Table 1 shows the characteristics of all participants. The study population included 426 participants, 253 HCs and 173 with T2DM. The mean age was 50.1 ± 9.0 years, and 51.6% of the population was female. Participants with higher GV were more prone to be older, more prone to be male, more prone to have a high level of education, more prone to have higher triglycerides, more prone to have higher ratio of total to HDL cholesterol, and more prone to have high blood pressure.

**Table 1.** Characteristics of the population

	HCs	Tertiles of visit-to-visit variability in HbA1c SD			*P*_trend_
		Low	Middle	High	
Range of HbA1c SD	−	0.1, 0.4	0.4, 1.1	1.1, 4.5	−
No. of participants	253	57	58	58	
Demographics					
Age (years)	49.0 ± 9.5	52.5 ± 7.6	50.8 ± 8.2	51.6 ± 8.1	.018
Female sex (%)	61.3	43.9	37.9	31.0	<.001
Education (years)	9.6 ± 4.6	10.8 ± 4.1	10.8 ± 4.1	11.4 ± 4.9	.002
Glucose metabolism					
Fasting glucose (mmol/L)	5.2 ± 0.5	7.3 ± 1.6	8.0 ± 1.7	9.1 ± 2.8	<.001
HbA1c (%)	5.7 ± 0.3	6.9 ± 1.2	7.6 ± 1.0	8.9 ± 1.6	<.001
Diabetes duration (years)	−	6.1 ± 4.8	7.1 ± 6.1	7.1 ± 5.6	−
Fasting insulin (mIU/mL)	10.2 ± 8.5	9.9 ± 5.6	10.4 ± 8.4	9.2 ± 12.9	.588
Cardiovascular risk factors					
BMI (kg/m^2^)	23.8 ± 3.3	24.0 ± 3.2	24.5 ± 3.7	24.0 ± 3.1	.302
Systolic BP (mmHg)	123.9 ± 18.0	128.7 ± 17.6	125.8 ± 14.2	129.4 ± 16.5	.031
Diastolic BP (mmHg)	81.3 ± 11.5	85.6 ± 10.1	82.6 ± 10.6	84.3 ± 10.5	.043
Hypertension (%)	15.8	35.1	32.8	34.5	<.001
Ratio of total to HDL cholesterol	3.8 ± 1.2	4.2 ± 1.1	4.4 ± 1.4	5.0 ± 1.8	<.001
LDL cholesterol (mmol/L)	3.2 ± 0.8	3.2 ± 0.9	2.8 ± 0.9	3.4 ± 1.0	.787
Triglycerides (mmol/L)	1.4 ± 1.2	2.0 ± 1.6	2.5 ± 2.6	2.4 ± 3.1	<.001
History of cardiovascular disease (%)	6.7	7.0	8.6	10.3	.328
Medication use					
Insulin + oral hypoglycemic drug (%)	−	35.1	48.3	56.9	−
Antihypertensive medication (%)	17.0	35.1	32.8	29.3	.004
Lipid-modifying medication (%)	11.1	33.3	41.4	41.4	<.001
Lifestyle factors					
Alcohol consumption (%)	17.8	33.3	25.9	20.7	.249
Smoking status (%), never/former/current	67.6/21.3/11.1	61.4/21.1/17.5	73.7/6.9/20.1	65.5/13.8/20.1	.250

Data are presented as mean ± SD for continuous variables and percentage for categorical variables.

Abbreviations: BMI, body mass index; BP, blood pressure; HbA1c, glycated hemoglobin; HC, healthy control; HDL, high-density lipoprotein. LDL, low-density lipoprotein.

After adjusting for age, gender, education, and mean HbA1c, participants with higher GV were more likely to have lower MMSE scores and were more likely to take longer time to complete the TMT-A (Table 2).

**Table 2.** Association between glucose variability in HbA1c SD and cognitive tests after adjustment for age, sex, education, and mean HbA1c

Cognitive tests	HCs	Tertiles of visit-to-visit variability in HbA1c SD			*P*_trend_
		Low	Middle	High	
MMSE	Ref	−0.70 (−1.47, 0.08)	−0.50 (−1.49, 0.48)	−1.68 (−2.89, −0.46)	.**014**
AVLT (immediate)	Ref	−0.43 (−2.15, 1.29)	1.01 (−1.27, 3.30)	1.67 (−1.09, 4.43)	.211
AVLT (5 minutes)	Ref	0.16 (−0.59, 0.92)	0.34 (−0.65, 1.34)	0.94 (−0.25, 2.13)	.203
AVLT (20 minutes)	Ref	−0.28 (−1.04, 0.48)	−0.26 (−1.27, 0.76)	1.11 (−0.08, 2.31)	.483
TMT-A	Ref	1.08 (−6.61, 8.76)	5.23 (−4.78, 15.23)	10.56 (−1.96, 23.08)	.**044**
DSST	Ref	−0.20 (−4.23, 3.83)	−0.64 (−5.70, 4.42)	0.25 (−5.97, 6.47)	.281
DST (forward)	Ref	0.16 (−0.32, 0.63)	−0.30 (−0.91, 0.30)	0.60 (−0.13, 1.32)	.667
DST (backward)	Ref	0.45 (−0.01, 0.91)	0.36 (−0.20, 0.91)	0.28 (−0.39, 0.94)	.365
GPT (R)	Ref	1.51 (−6.79, 9.80)	3.13 (−7.43, 13.69)	−0.13 (−13.28, 13.01)	.458
GPT (L)	Ref	−4.58 (−12.56, 3.41)	9.99 (−0.99, 20.96)	−5.09 (−17.89, 7.71)	.700

Boldface data represent *P* < .05. Abbreviations: AVLT, Auditory Verbal Learning Test; DSST, Digit Symbol Substitution Test; DST, Digital Span Test; GPT, Grooved Pegboard Test; HC, healthy control; MMSE, Mini-Mental State Test; TMT-A, Trail Making Test A.

### The Associations Between GV and Cortical Morphometry

**Figure 2.**
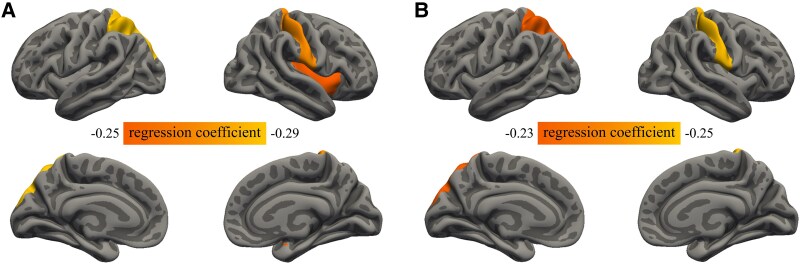
Cerebral morphology significantly associated with HbA1c SD and HbA1c CV after adjustment for Model III. (A) Cortical thickness of the left superior parietal cortex, right postcentral gyrus, and insula showed significant negative associations with HbA1c SD (*P*_FDR_ < 0.05). (B) Cortical thickness of the left superior parietal cortex and right postcentral gyrus demonstrated significant negative associations with HbA1c CV (*P*_FDR_ < 0.05). No significant associations were found between glucose variability and gray matter volume or cortical surface area after FDR correction.

In this study, we investigated the impact of each additional tertile of GV on brain morphometry. After adjusting for Model I, reduced cortical thickness in the right pars opercularis, postcentral gyrus, and superior temporal cortex was significantly associated with higher GV (*β* = −.14, −.19, −.14, respectively, see Table 3 & Supplementary Table S1 (10)). Additionally, gray matter volume atrophy in the right medial orbitofrontal cortex (−0.14, 95% CI: −0.21, −0.07) and pars opercularis (−0.17, 95% CI: −0.26, −0.08) was also significantly associated with higher GV. After further adjusting for additional covariates such as smoking status, alcohol consumption, and cardiovascular risk in Model II, the association between GV and cortical morphometry was strengthened. Reduced cortical thickness in the left inferior parietal cortex, superior parietal cortex, right postcentral gyrus, caudal middle frontal gyrus, bilateral pars opercularis, rostral middle frontal gyrus, superior frontal gyrus, and supramarginal gyrus was significantly associated with higher GV, with effect sizes ranging from −0.18 to −0.13 SD units per increase in GV level (Table 4 & Supplementary Table S2 (10)). Moreover, the association between gray matter volume in the right medial orbitofrontal cortex and GV remained significant (−0.13, 95% CI: −0.20, −0.05). Finally, after adjusting for mean HbA1c (Model III), we observed a decrease in the precision of the association between cortical thickness and GV in all significant brain regions from Model II (no evidence of collinearity between the GV and mean HbA1c was found), rather than a weakening of the association (Supplementary Table S3 (10)). Nevertheless, the association between cortical thickness and GV in the left superior parietal cortex (−0.29, 95% CI: −0.45, −0.13) and right postcentral gyrus (−0.28, 95% CI: −0.45, −0.12) remained significant, while the association in the right insula (−0.25, 95% CI: −0.41, −0.09) was significantly strengthened (Table 5 & Fig. 2). At the same time, after adjusting for mean HbA1c, the precision of the association between gray matter volume in the right medial orbitofrontal cortex and GV decreased (−0.13, 95% CI: −0.25, −0.02), and it was no longer significantly associated with GV after FDR correction. After adjustments across different models, no significant association between GV and cortical surface area was consistently observed. The associations between all pairwise GV and brain morphometry showed no significant differences between males and females (*P*_interaction_ > .10).

**Table 3.** Cerebral morphometry showing significant associations with the visit-to-visit glucose variability in SD of HbA1c after adjustment for Model I

Cerebral region	HCs	Tertiles of visit-to-visit variability in HbA1c SD			*P*_trend_
		Low	Middle	High	
**Cortical thickness, mm**					
Pars opercularis (R)	Ref	−0.05 (−0.08, −0.02)	−0.03 (−0.06, −0.01)	−0.04 (−0.07, −0.01)	.003
Postcentral gyrus (R)	Ref	−0.05 (−0.09, −0.01)	−0.05 (−0.08, −0.01)	−0.06 (−0.10, −0.02)	<.001
Superior temporal cortex (R)	Ref	−0.06 (−0.10, −0.03)	−0.03 (−0.07, −0.01)	−0.04 (−0.07, −0.01)	.003
**Cortical volume, cm^3^**					
Medial orbitofrontal cortex (R)	Ref	−0.13 (−0.25, −0.01)	−0.18 (−0.30, −0.06)	−0.21 (−0.34, −0.08)	<.001
Pars opercularis (R)	Ref	−0.33 (−0.49, −0.17)	−0.23 (−0.40, −0.06)	−0.28 (−0.45, −0.11)	<.001
**Cortical surface area, mm^2^**					
None	—	—	—	—	—
**Subcortical volume, cm^3^**					
Pallidum (R)	Ref	−0.04 (−0.09, 0.02)	−0.08 (−0.14, −0.03)	−0.09 (−0.15, −0.04)	<.001
**Brain volume, cm^3^**					
Total gray matter	Ref	−5.72 (−12.03, 0.59)	−8.03 (−14.25, −1.81)	−9.42 (−16.03, −2.81)	<.001

Model I adjusted for age, sex, education, and intracranial volume.

**Table 4.** Cerebral morphometry showing significant associations with the visit-to-visit glucose variability in SD of HbA1c after adjustment for Model II

Cerebral region	HCs	Tertiles of visit-to-visit variability in HbA1c SD			*P*_trend_
		Low	Middle	High	
**Cortical thickness, mm**					
Inferior parietal cortex (L)	Ref	−0.04 (−0.06, −0.01)	−0.02 (−0.05, 0.01)	−0.03 (−0.06, −0.01)	.007
Superior parietal cortex (L)	Ref	−0.03 (−0.06, 0.01)	−0.03 (−0.05, −0.01)	−0.04 (−0.07, −0.01)	<.001
Caudal middle frontal gyrus (R)	Ref	−0.03 (−0.06, −0.01)	−0.03 (−0.06, 0.01)	−0.05 (−0.08, −0.01)	.002
Postcentral gyrus (R)	Ref	−0.05 (−0.09, −0.01)	−0.04 (−0.08, −0.01)	−0.05 (−0.09, −0.01)	<.001
Pars opercularis (L)	Ref	−0.06 (−0.09, −0.03)	−0.02 (−0.05, 0.01)	−0.03 (−0.07, 0.01)	.009
Pars opercularis (R)	Ref	−0.06 (−0.09, −0.03)	−0.03 (−0.06, 0.01)	−0.04 (−0.07, −0.01)	.007
Rostral middle frontal gyrus (L)	Ref	−0.06 (−0.08, −0.03)	−0.03 (−0.06, −0.01)	−0.04 (−0.07, −0.01)	<.001
Rostral middle frontal gyrus (R)	Ref	−0.04 (−0.06, −0.01)	−0.03 (−0.05, −0.01)	−0.04 (−0.06, −0.01)	.004
Superior frontal gyrus (L)	Ref	−0.05 (−0.08, −0.02)	−0.03 (−0.06, 0.01)	−0.04 (−0.07, −0.01)	.003
Superior frontal gyrus (R)	Ref	−0.04 (−0.07, −0.01)	−0.03 (−0.06, −0.01)	−0.04 (−0.07, −0.01)	.002
Supramarginal gyrus (L)	Ref	−0.05 (−0.08, −0.02)	−0.02 (−0.05, 0.01)	−0.03 (−0.06, −0.01)	.007
Supramarginal gyrus (R)	Ref	−0.02 (−0.06, 0.01)	−0.02 (−0.05, 0.01)	−0.05 (−0.08, −0.02)	.001
**Cortical volume, cm^3^**					
Medial orbitofrontal cortex (R)	Ref	−0.11 (−0.24, 0.01)	−0.18 (−0.31, −0.05)	−0.17 (−0.31, −0.03)	<.001
**Cortical surface area, mm^2^**					
None	—	—	—	—	—
**Subcortical volume, cm^3^**					
Pallidum (R)	Ref	−0.03 (−0.08, 0.03)	−0.09 (−0.14, −0.03)	−0.10 (−0.15, −0.04)	<.001
**Brain volume, cm^3^**					
Total gray matter	Ref	−5.21 (−11.65, 1.23)	−5.94 (−12.37, 0.50)	−6.83 (−13.77, 0.11)	.006

Model II adjusted for age, sex, education, intracranial volume, body mass index, smoking status, alcohol consumption, the ratio of total to high-density lipoprotein cholesterol, serum triglycerides, and hypertension. Abbreviation: HC, healthy control.

**Table 5.** Cerebral morphometry showing significant associations with the visit-to-visit glucose variability in SD of HbA1c after adjustment for Model III

Cerebral region	HCs	Tertiles of visit-to-visit variability in HbA1c SD			*P*_trend_
		Low	Middle	High	
**Cortical thickness, mm**					
Superior parietal cortex (L)	Ref	−0.04 (−0.07, 0.01)	−0.05 (−0.09, −0.01)	−0.09 (−0.15, −0.04)	<.001
Postcentral gyrus (R)	Ref	−0.05 (−0.10, −0.01)	−0.09 (−0.15, −0.03)	−0.09 (−0.17, −0.02)	<.001
Insula (R)	Ref	−0.12 (−0.16, −0.08)	−0.07 (−0.13, −0.01)	−0.07 (−0.14, −0.01)	.002
**Cortical volume, cm^3^**					
None	—	—	—	—	—
**Cortical surface area, mm^2^**					
None	—	—	—	—	—
**Subcortical volume, cm^3^**					
None	—	—	—	—	—
**Brain volume, cm^3^**					
Total gray matter	Ref	−5.89 (−14.00, −2.23)	−17.16 (−27.48, −6.84)	−10.61 (−23.34, 2.12)	.008

Model III adjusted for age, sex, education, intracranial volume, body mass index, smoking status, alcohol consumption, the ratio of total to high-density lipoprotein cholesterol, serum triglycerides, hypertension and mean HbA1c. Abbreviation: HC, healthy control.

### The Associations Between GV and Subcortical Regions and Brain Volume

We further investigated the association between each additional tertile of GV and changes in subcortical regional volumes and total brain volume. We found that, after FDR correction in Model I, the gray matter volume of the right pallidum (−0.16, 95% CI: −0.23, −0.08, *P*_FDR_ = .001) and total gray matter volume (−0.07, 95% CI: −0.11, −0.03, *P*_FDR_ = .002) were negatively associated with GV (Table 3). In Model II, the associations between GV and the right pallidum (−0.16, 95% CI: −0.24, −0.08, *P*_FDR_ = .001) and total gray matter volume (−0.06, 95% CI: −0.10, −0.02, *P*_FDR_ = .011) remained significant (Table 4). Finally, after adjusting for mean HbA1c (Model III), the association between total gray matter volume and GV was strengthened but with decreased precision (−0.09, 95% CI: −0.16, −0.02, *P*_FDR_ = .016). The association between the gray matter volume of the right pallidum and GV weakened slightly and showed decreased precision (−0.14, 95% CI: −0.27, −0.02, *P*_FDR_ = .189), and it was no longer significantly associated with GV after FDR correction (Table 5).

### Sensitivity Analysis

The results showed that our findings remained stable under several circumstances. First, after excluding patients with intracranial volumes beyond 3 SDs, the results of the sensitivity analyses were consistent with our main findings (Supplementary Tables S4-S6 (10)). Second, using the CV as an indicator of HbA1c variability did not significantly alter our results (Supplementary Tables S7-S8 (10)), but the association between cortical thickness in the right insula and GV was no longer significant after adjusting for mean HbA1c (Supplementary Table S9 (10)). Finally, after excluding prediabetic participants from the healthy control group, the direction of the association between GV and brain morphometric measures remained consistent with the main analysis, and the effect size was slightly enhanced (Supplementary Tables S10-S12 (10)).

## Discussion

This study investigated the association between visit-to-visit GV and cognitive function in patients with T2DM, while comprehensively mapping the associations between GV and brain morphometry using multi-scale MRI data. First, we found that higher GV was associated with poorer global cognitive performance and executive function. Secondly, through structural MRI, we observed that higher GV was correlated with cortical thinning and reduced total gray matter volume, independent of sociodemographic factors, cardiovascular risk factors, and mean HbA1c. The strength of these associations was generally comparable to that of the association with mean HbA1c during the study period. Sensitivity analyses did not significantly alter our findings, indicating that our results are robust.

In this study, we observed significant associations between GV and cognitive function. For example, for every 1-tertile increase in GV, MMSE decreased by 0.37 points (95% CI: −0.66, −0.08) and TMT-A completion time increased by 3.04 seconds (95% CI: 0.08, 6.00). These associations suggest that GV may affect normal cognitive function in T2DM patients (21). A longitudinal study showed that participants in the quartile with the highest HbA1c variability in T2DM patients had significantly worse rates of memory decline and executive function decline in 2 different cohorts (22). Another retrospective study reported that cognitive function in middle-aged and elderly T2DM patients was affected by long-term glycemic variability, and individuals with higher glycemic variability had poorer cognitive function (23). Previous findings are consistent with current results, despite the variation in cognitive tests used. In addition, we also found positive associations between visit-to-visit GV and cardiovascular risk factors, which were consistent with previous findings (24-26). These suggest that homeostasis control of glucose impacts the normal functioning of both the cardiovascular system and the CNS.

After fully adjusting for confounders, higher GV was significantly associated with cortical thinning in the left superior parietal cortex, right postcentral gyrus, and insula. These regions overlap with areas previously reported to exhibit brain atrophy in patients with T2DM (27, 28). Thus, the cortical thinning seems likely to be the main manifestation of GV-related abnormal brain alterations. Given that the size, arrangement, and density of glia, neurons, and nerve fibers are reflected in cortical thickness (29), the observed reduction in cortical thickness may imply that there is neuronal death in the identified areas. In addition to affecting cortical thickness, total gray matter was also found to be negatively correlated with GV. However, after adjusting for mean HbA1c, we did not find any significant associations between GV and cortical volume or surface area. Due to the cortical thickness, gray matter volume, and cortical surface area are influenced by genetics and histology, and the variability of cortical surface area and gray matter volume measurements in the general population is higher than that of cortical thickness (30), which may be the main reason why the differences between groups of cortical surface area and gray matter volume are less sensitive compared to cortical thickness.

In sensitivity analysis, when using the CV of HbA1c to represent glycemic variability, the direction of the association between GV and cortical thickness remained unchanged after fully adjusting for confounders (Model III), but the effect size slightly decreased. Additionally, the association between the thickness of the right insular cortex and GV was no longer significant. The observed changes may be related to over-adjustment in the model. Since CV normalizes variability by dividing the SD by the mean, it partially removes the influence of mean HbA1c. Further adjustment for mean HbA1c in Model III might have diluted the independent effect of CV on cortical thickness. Moreover, certain brain regions (such as the right insula) may be more sensitive to absolute fluctuations in blood glucose (reflected by SD) rather than relative fluctuations (reflected by CV). For example, SD directly reflects the magnitude of blood glucose fluctuations, which may more readily induce oxidative stress or microvascular damage, whereas the “relative fluctuations” captured by CV may not fully account for such biological effects. A review on the adverse effects of GV also indicates that different measurement methods of GV have independent predictive value for macrovascular and microvascular complications in diabetes (31). Future studies are needed to validate these observed associations.

A number of mechanisms could help explain the observed associations between GV and cognitive impairment and brain morphometric abnormalities. GV is linked to increased generation of reactive oxygen species, resulting in vascular injury inside the CNS (7, 8, 32). An in vivo study involving people with diabetes and healthy subjects showed that fluctuations in blood sugar were more prone to produce the toxic effects of oxidative stress and endothelial dysfunction than stable blood sugar levels (33). Prior research has shown that blood glucose fluctuation can increase the expression of inflammatory mediators, such as IL-1a and TNF-a, and promote the apoptosis of hippocampal neurons (34). Inflammatory mediators can penetrate the blood-brain barrier and infiltrate the brain, causing further harm to neurons and glial cells (35, 36), which diminishes neurotrophic support and initiates many neurodegenerative pathways, finally culminating in brain atrophy and white matter hyperintensities (37). Hyperglycemia is accompanied by compensatory endogenous insulin overproduction, potentially resulting in hypoglycemic episodes or cerebral and peripheral insulin resistance (38). Damage to brain insulin receptors and signals may lead to neuronal apoptosis, abnormal brain energy metabolism, and microvascular disorders (39). Finally, neurodegeneration of particular brain areas linked to cognitive processes ultimately results in impaired cognitive function. Therefore, multiple secondary outcomes triggered by increased GV may independently contribute to diabetes-related brain damage, independent of average plasma glucose levels. Simultaneously, proactive glycemic control may provide additional benefits for brain and cognitive outcomes.

Strengths of this study include characterization of long-term GV with good reproducibility of sensitivity analyses; multidimensional MRI to comprehensively analyze the effects of GV on brain structure; appropriate statistical models; and extensive assessment of potential confounders. There were inevitably some limitations to this study. First, due to the observational methodology, associations can only be inferred from our findings, but reverse causality cannot be ruled out. Second, healthy controls in this study had only 1 HbA1c measurement, and linear trend analysis was performed on the assumption that healthy controls had minimal glucose variability. Considering that the pancreas function of healthy subjects is normal and can accurately regulate blood glucose in a timely manner, the hypothesis that healthy subjects have minimal glucose variability is valid. Third, due to the retrospective nature of HbA1c data acquisition from clinical databases, variations in follow-up duration across individuals were unavoidable when calculating GV. However, these results can reflect realistic clinical situations that can inform health care decisions. Finally, after controlling for numerous potential confounders, we could not eliminate the effect of residual or unmeasured variables, including physical activity and dietary habits.

## Conclusion

We found that higher visit-to-visit GV in HbA1c was associated with poorer performance in global cognition and executive function. Furthermore, structural MRI revealed that reduced cortical thickness and decreased total gray matter volume were associated with higher GV, independent of mean HbA1c levels. In summary, these findings underscore the adverse impact of long-term GV on cognition and brain structure in patients with T2DM, providing deeper insights into the potential mechanisms underlying diabetes-related brain damage. Further prospective studies are needed to determine whether reducing GV could mitigate cognitive decline and brain atrophy in patients with T2DM.

## Data Availability

Data are available from the authors upon reasonable request.

## Abbreviations

CNS: central nervous system
CV: coefficient of variation
FDR: False Discovery Rate
GV: glucose variability
HbA1c: glycated hemoglobin
HC: healthy control
HDL: high-density lipoprotein
MMSE: Mini-Mental State Test
MRI: magnetic resonance imaging
T2DM: type 2 diabetes mellitus
TMT-A: Trail Making Test A
